# Comparative genomics and phylogenetic analysis of three Malvaceae species on the basis of chloroplast genomes

**DOI:** 10.3389/fpls.2026.1860109

**Published:** 2026-08-31

**Authors:** Xiangbo Liu, Jiaxuan Song, Ning Wang, Shuxiang Feng, Huiying Wang, Lingli Li, Xuan Ye, Yinran Huang, Yongtan Li, Yichao Liu

**Affiliations:** 1 College of Forestry, College of Landscape and Tourism, Hebei Key Laboratory of Floral Biological Breeding, Hebei Agricultural University, Baoding, China; 2 Key Laboratory of National Forestry and Grassland Administration on Colorful Tree, Hebei Agricultural University, Baoding, China; 3 Hebei Forestry and Grassland Science Research Institute, Shijiazhuang, China

**Keywords:** chloroplast genome, hypervariable regions, Malvaceae, phylogenetic analysis, phylogenomics

## Abstract

**Introduction:**

The Malvaceae family shows rich species diversity and has substantial economic and medicinal value. However, the frequent interspecific hybridization among members of this family has resulted in confused phylogenetic relationships among the groups, limiting the usefulness of traditional classification methods.

**Methods:**

This study aimed to investigate the phylogenetic relationships among selected taxa of Malvaceae by evaluating 23 chloroplast (CP) genomes, including three newly assembled CP genomes. Among these three genomes, the CP genome of *Hibiscus schizopetalus* L. was reported for the first time, while the CP genomes of Alcea rosea L. and *Hibiscus grewiifolius* L., which have been deposited in NCBI, were re-analyzed here alongside newly generated data for comparative purposes. In addition, 20 downloaded CP genomes encompassing 13 genera were analyzed using SNPs in whole CP genomes data.

**Results:**

The results showed that the genomes ranged from 160,403 to 161,978 base pairs in length and consisted of small single copies (SSCs) and large single copies (LSCs) separated by two inverted repeat sequences (IRs), forming a typical quadripartite circular structure. The entire genome sequence showed relative conservation across species in terms of structure, GC content, codon usage, and gene composition. The mutation sites were mainly located in the LSC and SSC regions, and the variability in the non-coding regions was higher than that in the coding regions. The nucleotide polymorphism (Pi) analysis identified the non-coding regions such as *ndhF-rpl32* and *psbZ-trnG* as high variable hotspots. A maximum likelihood phylogenetic tree was constructed based on SNPs in whole CP genomes data. The phylogenetic analysis divided these 23 species into five highly supported clades. It also revealed a close sister-group relationship between *Abelmoschus* and *Hibiscus* species, suggesting that Hibiscus may have a separate lineage from okra species.

**Discussion:**

In conclusion, the increasing availability of CP genome resources will enhance our understanding of the classification and evolutionary patterns of the Malvaceae family. The development of molecular markers will provide important molecular evidence for precise identification and classification revision of plants in this family.

## 1 Introduction

The Malvaceae family contains approximately 4,225 species. However, according to the NCBI database, complete chloroplast (CP) genome sequences are available only for over 130 species, including species such as *Hibiscus taiwanensis*, *Theobroma cacao*, and *Abelmoschus sagittifolius (*Wu et al., 2023; Wu, 2025). Plants of this family are widely distributed in tropical, subtropical and temperate regions worldwide, and can adapt to a variety of habitats ranging from forests and grasslands to arid areas and coastal zones (T.A.P.J.B.J.o.t.L.S. Group, 2016). The members of this family include herbaceous plants, shrubs and even trees, and their forms are diverse, with most flowers having five petals and displaying bright colors. The leaves are alternate, single or divided. The leaf veins are usually palmate and have stipules. The fruit is a capsule, often with several segments splitting, and rarely berry-like. The seeds are kidney-shaped or oblong, covered with hair or smooth and hairless, with endosperm. The cotyledons are flat, folded or spiral (本.J.f.o.c. 编委会, 2013). These plants are of great importance in terms of there ornamental (Barrett et al., 2025), economic (Colli-Silva et al., 2025), edible (Tao et al., 2025) and medicinal (Zini and Lattar, 2025; Wu et al., 2025) value.

Although members of the Malvaceae family are widely used in traditional medicine and modern research, more in-depth studies on specific species are required (M.I. China, 2014; Wang et al., 2023; Liu et al., 2025; Yang et al., 2026). These plants contain multiple categories of chemical components, including various bioactive compounds such as carotenoids, phenolic acids, flavonoids, coumarins, alkaloids, lignans, glycosides, sterols, terpenes, and polysaccharides (Sameh et al., 2024). *Alcea rosea* L. belongs to the genus Alcea, commonly known as hollyhock (Mortas, 2023). It is native to the southwestern region of China and is widely distributed throughout China. It can be found in the eastern, central, northern regions, Sichuan, Guizhou, and southern parts of China. It is also widely cultivated all over the world. The plant is tall, reaching up to 2m. The stems are upright and densely covered with star-shaped hair and bristles. The leaves are nearly heart-shaped, with sparse star-shaped hairs on the upper surface and star-shaped long hairs on the lower surface. The flower clusters are terminal single-petaled or double-petaled with colors such as purple, pink, red, and white. The fruits are disc-shaped, covered with short hairs and have longitudinal grooves. The seeds are kidney shaped. The flowering period is from February to August. In traditional medicine, the entire plant of *A*. *rosea* can be used as a medicinal ingredient. It contains flavonoids, phenolic acids, amino acids, fatty acids, anthocyanins, polysaccharides and more (Kim et al., 2018). The flowers and young leaves of the hibiscus plant are rich in mucilage, flavonoids, and phenolic acids, and exhibit anti-inflammatory, analgesic, and expectorant properties. The young leaves can also be eaten as a vegetable, demonstrating the characteristic “medicinal and edible” nature of plants in this family (本.J.f.o.c. 编委会, 2013; Hanif et al., 2019). *Hibiscus schizopetalus* L. is an evergreen shrub of the genus Hibiscus. It is commonly known as lantern flower and is native to the tropical regions of East Africa. It is cultivated in Taiwan, Fujian, Guangdong and other places in China. It can grow up to 3m tall, with the small branches often drooping; the leaves are elliptical or long-rectangular, with the apex pointed or short-acuminate, and the base wedge-shaped; the pedicels are slender, hairless or with fine hairs, and have a node in the middle; the capsule is long cylindrical; the flowering period is throughout the year (本.J.f.o.c. 编委会, 2013). This species contains 48 types of secondary metabolites such as phenolic acids, flavonoids, anthocyanins, fatty acids and fatty amides (El-Shiekh et al., 2020; El-Din et al., 2024). The leaves and roots of *H*. *schizopetalus* are used in traditional medicine. The plant is considered pungent and cooling and is used to clear heat, detoxify the body, and treat conditions such as axillary sores and abdominal distension associated with qi stagnation (Wong et al., 2016). Due to the extremely unique shape and excellent ornamental value of the flowers, this plant is suitable for cultivation and display in parks, courtyards, residential areas, etc., and can also be used for hedging (Seth, 2015). *Hibiscus grewiifolius* L. belongs to the genus Hibiscus and is a small evergreen tree. It gets its name because its leaves resemble those of the camphor tree. It is native to Hainan, China. It grows on mountainous areas and forests at an altitude of 2000m. It is also distributed in Vietnam, Laos, Thailand, Myanmar, and Indonesia. It can reach a height of up to 7m. The small branches are cylindrical, pale gray-white, smooth, and hairless or with extremely fine hair. The leaves are papery to nearly leather and ovate to elliptic-lanceolate, with a short acute tip at the apex. The base is obtuse to broad cuneate the basal veins are raised on the underside the petiole is sparsely covered with short hairs; and the stipules are small. The flowers are large and yellow. The fruits are solitary in the axils of the upper leaves; the fruit stalk is smooth and hairless; the bracteoles are linear; the perianth long and round-needle-shaped; the capsule is oval and hairless; the seeds are present in each fruit, kidney-shaped, and the back is densely covered with fuzz. The flowers are large and colorful, and the flowering period lasts for half a year. The plants are easy to propagate, making them excellent ornamental plants for gardens with high ornamental value (Zheng, 1998; 本.J.f.o.c. 编委会, 2013). They are rich in phytochemical components such as antioxidants, phytosterols, saponins, lignans, volatile oils, glycosides, and anthocyanins (Kwon et al., 2023).

Malvaceae is an important family for studying plant phylogeny, adaptive evolution and genomics due to the rich variety of species, wide distribution and diverse characteristics (Huang et al., 2024; Zhang et al., 2025). However, the phylogenetic relationships of various subgroups within some genera in this family remain topics of debate. In particular, the systematic position of the genus *Hibiscus* and the issue of its boundaries with related groups are the most prominent (Cvetkovi et al., 2021; Liu et al., 2026). More recently, with the widespread adoption of sequencing technologies, the CP genome has emerged as a powerful tool for resolving both deep and shallow phylogenetic relationships due to its maternally inherited nature, conserved structure, and moderate evolutionary rate (Odintsova and Yurina, 2003; Li L. et al., 2025). In comparison with nuclear genomes, CP genomes show significant advantages such as high copy number, ease of assembly, and low recombination rate, and can provide more reliable phylogenetic information, especially information regarding the systematic position of the *Hibiscus* genus and its boundaries with related groups, at a lower cost. Although a large amount of CP genome data of leguminous plants has been accumulated in public databases (Won, 2009), most of the existing studies have focused on a few species or specific branches, lacking comprehensive sampling and holistic analysis of multiple genera within a family, especially those controversial key groups (Conover et al., 2019; Kayal et al., 2024). Furthermore, most studies have not fully incorporated closely related outgroups in comparative analyses, resulting in a relatively narrow phylogenetic framework. This limitation significantly reduces the robustness of phylogenetic inferences, the support values of key nodes, and the accuracy of divergence time estimates.

Therefore, this study aimed to: (1) Compare the genomic structures and analyze the evolution of selected taxa of Malvaceae by supplementing the CP genome data of key species, including *A*. *rosea*, *H*. *schizopetalus*, and *H. grewiifolius*, thereby enriching the genomic resources of the Malvaceae family. Among these, the CP genome of *H*. *schizopetalus* is reported for the first time, whereas the CP genomes of *A*. *rosea* and *H*. *grewiifolius* which have been deposited in NCBI, were re-analyzed here alongside newly generated data for comparative purposes. Enrich the genomic resources of the Malvaceae family. (2) Integrate the genomic data of 17 species from the same family available in the public database. To enhance the robustness of the phylogenetic inference, three closely related outgroups were additionally included, resulting in an evolutionary framework comprising 23 species. Comparative analyses of CP genome structural variations and evolutionary patterns were then performed. (3) Investigate the phylogenetic relationships among the selected taxa of Malvaceae in a broader phylogenetic context and explore the evolutionary mechanisms underlying variations in CP genome structure. (4) To provide more precise genomic support for systematic classification revision, phylogenetic analysis, ecological adaptation and functional gene mining of the selected taxa of Malvaceae.

## 2 Materials and methods

### 2.1 Sample collection, DNA extraction and sequencing

In this study, all plant materials of *A. rosea*(longitude 114°47’, latitude 38°05’), *H*. *schizopetalus* (longitude 114°47’, latitude 38°05’), and *H*. *grewiifolius* (longitude 114°47’, latitude 38°05’) were collected from the Forestry Science Research Institute of Hebei Province (longitude 114°47’, latitude 38°05’) (**Supplementary Table 1**). Fresh leaves of the three Malvaceae species were immediately frozen in liquid nitrogen after collection and stored at -80°C, and their total DNA was extracted using the Plant Genome DNA Extraction Kit (TianGen Biotechnology Beijing Co., China). Subsequently, a sequencing library with an average insert fragment length of 350 bp was constructed, and dual-end sequencing was performed in accordance with the manufacturer’s protocol of the Illumina NovaSeq platform, yielding 150 bp read lengths. The sample data volumes of *A. rosea*, *H*. *schizopetalus*, and *H*. *grewiifolius* were 4.1, 4.6, and 4.1 G respectively, and the average coverage of the CP genome exceeded 100×.

### 2.2 Genome assembly and annotation

The quality of raw sequencing data was assessed by FastQC, and Trimmomatic software (Bolger et al., 2014) was used to eliminate adapters and low-quality reads. The SOAPnuke software (Luo et al., 2015) (SOAPdenovo2 https://sourceforge.net/projects/soapdenovo2/files/GapCloser/) was used to filter the raw data, removing sequences containing more than 5% N bases, sequences with quality scores ≤ 50%, and sequences contaminated by adapters, there by yielding high-quality sequences and improving the reliability of data analysis. The CP genome sequences of Malvaceae downloaded from the National Center for Biotechnology Information of the United States (NCBI, https://www.ncbi.nlm.nih.gov/) were compared (**Supplementary Table 2**), and the aligned read fragments were extracted using Bowtie 2 (Langmead and Salzberg, 2012). The extracted sequence was processed using NOVOPlasty 4.2 (Dierckxsens et al., 2017), spliced into the CP genome, and the gaps were filled using GapCloser 1.12 (Luo et al., 2012) to obtain the complete sequence. The assembled genome was annotated by CPGAVAS2 (Shi et al., 2019) and then manually corrected. Identification of tRNA genes was performed using the tRNAscan-SE software (Lowe and Chan, 2016), and the CP genome map was generated using the Organellar Genome DRAW tool (Greiner et al., 2019).

### 2.3 Shrinkage and expansion of IR region

To facilitate comparisons among various species, the CP genomes of 12 species from the NCBI database were downloaded (**Supplementary Table 2**). On the basis of annotation and analysis performed using IRscope V0.1 (Amiryousefi et al., 2018) software, we analyzed and compared the four regions of the CP genomes of plants from Malvaceae. By visualizing the shrinkage and expansion of the inverted repeat (IR) boundary between the four regions of the genome (large single copy [LSC]/inverted repeat b [IRb]/small single copy [SSC]/inverted repeat a [IRa]), the diversity of the CP genome structure of different plants was characterized and the plants were preliminarily classified.

### 2.4 Long repeat and simple-sequence repeat analysis

MISA software (Sebastian et al., 2017) was used to identify simple sequence repeats (SSRs) in the CP genomes of the Hibiscus genus and its related groups. The SSRs located in the coding regions were distinguished from those located in the intergenic spacer (IGS) regions. Using the MISA-web server (Guo et al., 2017), the minimum repeat numbers for mononucleotide, dinucleotide, trinucleotide, tetranucleotide, pentanucleotide, and hexanucleotide motifs were set to 8, 4, 4, 3, 3, and 3, respectively. Using REPuter, long repetitive sequences with forward, reverse, complementary and palindromic fragments were located using the following parameters minimum repeat length 30 bp; and Hamming distance 3 (Kurtz et al., 2001). To distinguish SSRs in coding regions from those in IGS regions, we re-performed SSR detection using the standalone MISA program (embedded in the cpstools package) with adjusted thresholds: mono- ≥ 10, di- ≥ 6, tri- ≥ 5, tetra- ≥ 4, penta- ≥ 4, and hexa- ≥ 4.

### 2.5 Collinearity analysis

Genome-rearrangement analysis of 15 species was performed using the progressive Mauve method in Mauve software (version 2.3.1) (Darling et al., 2004; Darling et al., 2010).

### 2.6 Genome linear structure and nucleotidepolymorphism analysis

Comparative analysis of the complete CP genome sequences of 15 Malvaceae species was performed using the mVISTA online program (https://genome.lbl.gov/vista/) with the Shuffle-LAGAN alignment model (Mayor et al., 2000; Frazer et al., 2004). DNASP 6 was used to calculate the nucleotide diversity (Pi) values of the coding and intergenic regions. In addition to the newly obtained genomic sequences in the experiment, 20 CP genomes (**Supplementary Tables 1, 2**) of Malvaceae plants were downloaded from NCBI for phylogenetic analysis (Librado and Rozas, 2009). To evaluate the phylogenetic utility of hypervariable regions, individual and concatenated datasets of these regions were used to reconstruct maximum likelihood (ML) trees using FastTree with 1,000 bootstrap replicates (Price et al., 2009; Price et al., 2010). The resulting topologies and node support values were compared against the ML tree inferred from the SNPs in whole CP genomes data.

### 2.7 Codon usage bias and Ka/Ks analysis

To enable direct comparison of relative synonymous codon usage (RSCU) values across species, a comparative analysis was performed using the online platform RSCU-Plot (Akrami et al., 2025; Diani Gohar and Soorni, 2025; Hejazi et al., 2025; Soorni and Golchini, 2025; Zhang and Feng, 2025). The effective number of codons (ENc), which ranged from 20 to 61, was used as an indicator to measure the codon usage preference of the entire genome (Wright, 1990). To investigate the factors influencing the usage pattern of genetic codons and the relationship between base composition and codon-usage bias, the ENC plot graph was plotted using the GC3 value as the x-axis and the ENC value as the y-axis. To assess the difference between the observed value (ENCobs) of the effective codon count and the expected value (ENCexp), the frequency of the ENC ratio was calculated and these differences were quantified (Wright, 1990). The standard curve in the figure was obtained using the following equation: $\text{ENC}=2+\frac{9}{\text{F}2}+\frac{1}{\text{F}3}+\frac{5}{\text{F}4}+\frac{3}{\text{F}6}$. The Ka/Ks ratio was determined using the KaKs_Calculator tool (Zhang, 2022).

### 2.8 Phylogenetic analysis

On the basis of the data collected in this study, the CP genome sequences of 22 species of the Malvaceae family were analyzed. The CP genome of *Ricinus communis* L. was regarded as an outgroup. The MAFFT V7.310 algorithm was used to extract and compare common protein-coding genes (Katoh and Standley, 2013), and TrimAl V1.4 (Capella-Gutiérrez et al., 2009) was used for pruning. The ML method was used to construct the phylogenetic tree. The software used was PhyML v3.0, and the bootstrap value was 1,000 (Alexandros and Bioinformatics, 2014), to resolve the interspecific relationships among the sampled taxa.

## 3 Results

### 3.1 Basic characteristics of CP genomes in three species of the Malvaceae

**Figure 1:**
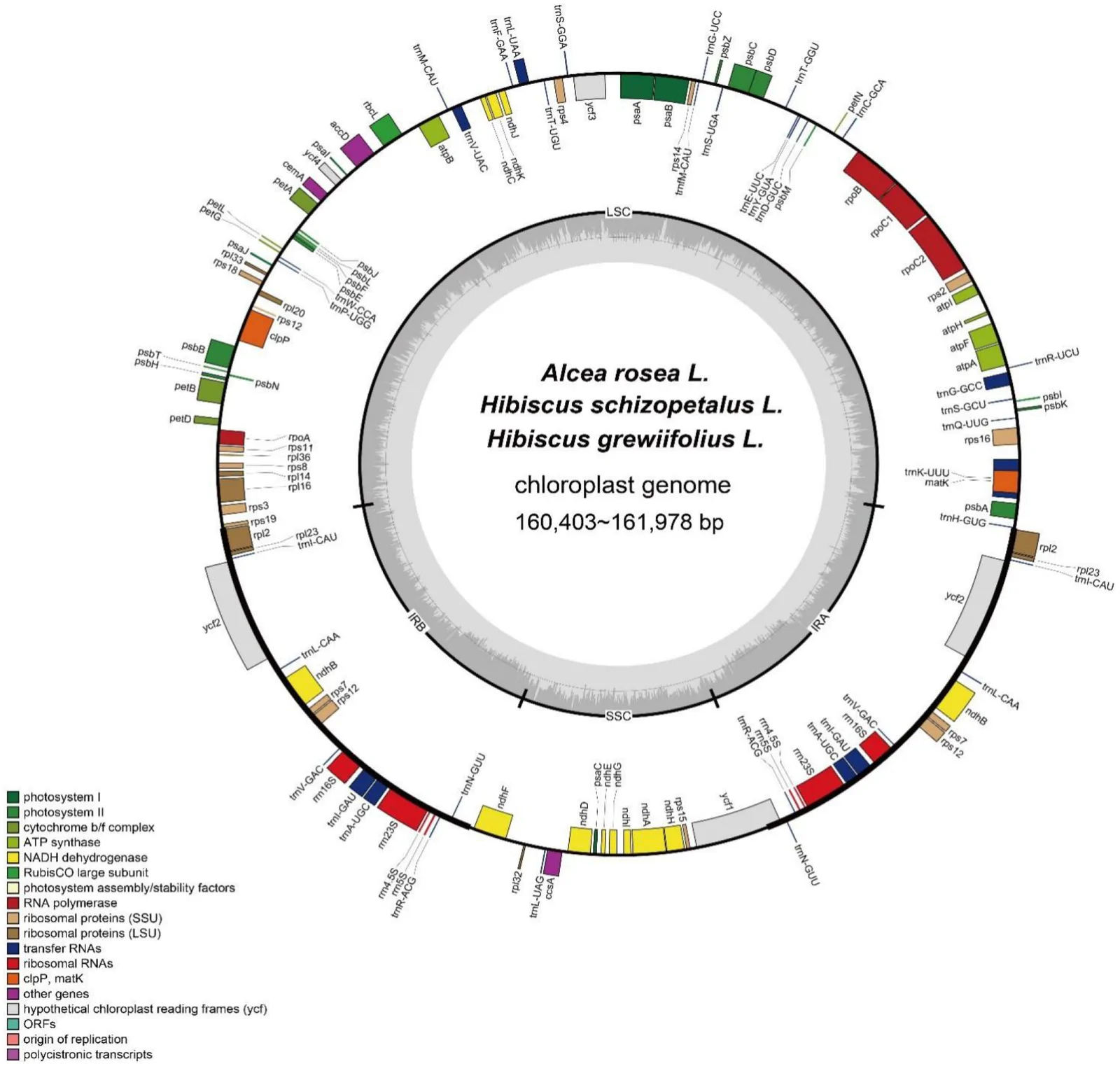
CP genome map of three Malvaceae species in this study. The dark gray in the inner circle corresponds to the GC content, and light gray represents AT content.

The CP genomes of three Malvaceae species, including *A. rosea*, *H. schizopetalus*, and *H. grewiifolius*, were successfully assembled (**Figure 1**). The comparative analysis indicated that the gene sequences, contents, and cluster organizations of the three species were highly conserved. Their lengths were 160,403, 160,493 and 161,978 bp respectively, and the GC content ranged from 36.81% to 37.04% (**Supplementary Table 1**). The CP genomes exhibited a typical quadripartite circular structure, consisted of an LSC region and an SSC region separated by a pair of IRs (IRa and IRb). The GC content peaked in the IR region (42.66-42.94%), followed by the LSC region (34.65-34.95%) and the SSC region (30.84-31.30%). The size of the LSC region accounted for more than 50% of the entire genome length. IRa and IRb are repetitive structures that encode the same gene in opposite directions. All genomes contained 114 unique genes, including four rRNA genes, 30 tRNA genes and 80 protein-coding genes. Due to the presence of IR regions, some genes had two copies, so the total number of genes in each genome was 130 (**Supplementary Table 3**).

### 3.2 Comparison of IR/single-copy boundaries and analysis of genomic length variations

**Figure 2:**
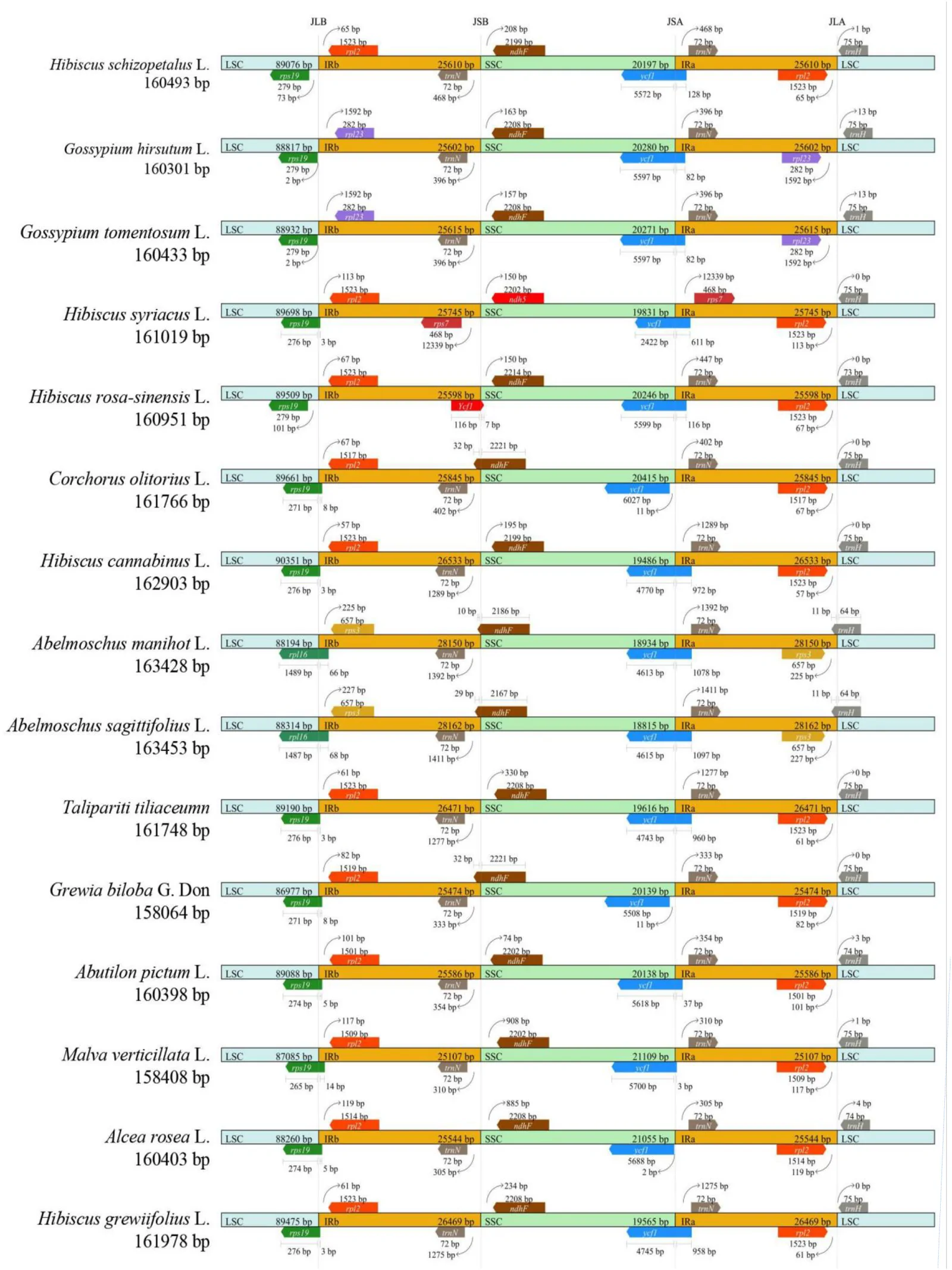
Comparison of the LSC, IR, and SSC border regions among 15 Malvaceae CP genomes. JLB, JSB, JSA, and JLA are the junction boundaries of LSC/IRb, SSC/IRb, SSC/IRa, and LSC/IRa, respectively.

To investigate the patterns in the CP genomes of the selected taxa of Malvaceae, we conducted a comparative analysis of the three newly sequenced Malvaceae species in this study and 12 representative species downloaded from the NCBI database (**Supplementary Table 2**). We precisely compared the boundaries of the IR and single-copy (SC) regions of these 15 Malvaceae species (**Figure 2**). Although the arrangement of boundary genes was highly conserved within families, significant expansion and contraction were observed at the boundary between the IR and SC (IR/SC) regions. Their lengths ranged from 25,107 bp to 28,162 bp, with a difference of 3,055 bp. This was the main reason for the variations in the total genome length. Which were based on the distribution of the key boundary genes *ndhF* and *ycf1*. At the junction of the IRb and SSC regions (JSB), the IRb region ended upstream of the *ndhF* gene with an intergenic sequence of 9–28 bp in 11 species, namely, *A*. *rosea*, *G*. *hirsutum*, *Gossypium tomentosum* L., *H*. *cannabinus*., *H*. *rosa*-*sinensis*, *H*. *schizopetalus*., *H*. *grewiifolius*, *H*. *syriacus*, *T*. *tiliaceumn*, *Abutilon pictum* L., and *M*. *verticillata*. In four species, namely, *A*. *manihot*, *Abelmoschus sagittifolius* L., *Grewia biloba* G. Don, and *Corchorus olitorius* L., the IRb boundary extended to the SSC region and entered the 10–32 base pairs of the *ndhF* coding region. At the JSB, the boundary relationship of the *ycf1* gene was more diverse and exhibited greater variation. In three species, namely, *A*. *rosea*, *G*. *biloba* G. Don, and *C*. *olitorius*, the IRa region boundary was located upstream of the *ycf1* gene, with short spacers of 2–11 bp. In the remaining 12 species, the IRa region boundary extended into the 5’ coding region of the *ycf1* gene, with an overlap length ranging from 74 bp to 908 bp, indicating that this boundary had higher plasticity. In summary, the dynamic evolution of the IR/SSC boundary was a central feature of CP genome structural variation in Malvaceae, and boundary expansion on the *ycf1* side (maximum overlap 908 bp) was much more pronounced than that on the *ndhF* side (maximum overlap 32 bp).

### 3.3 Long repetitive sequences and SSR analysis

**Figure 3:**
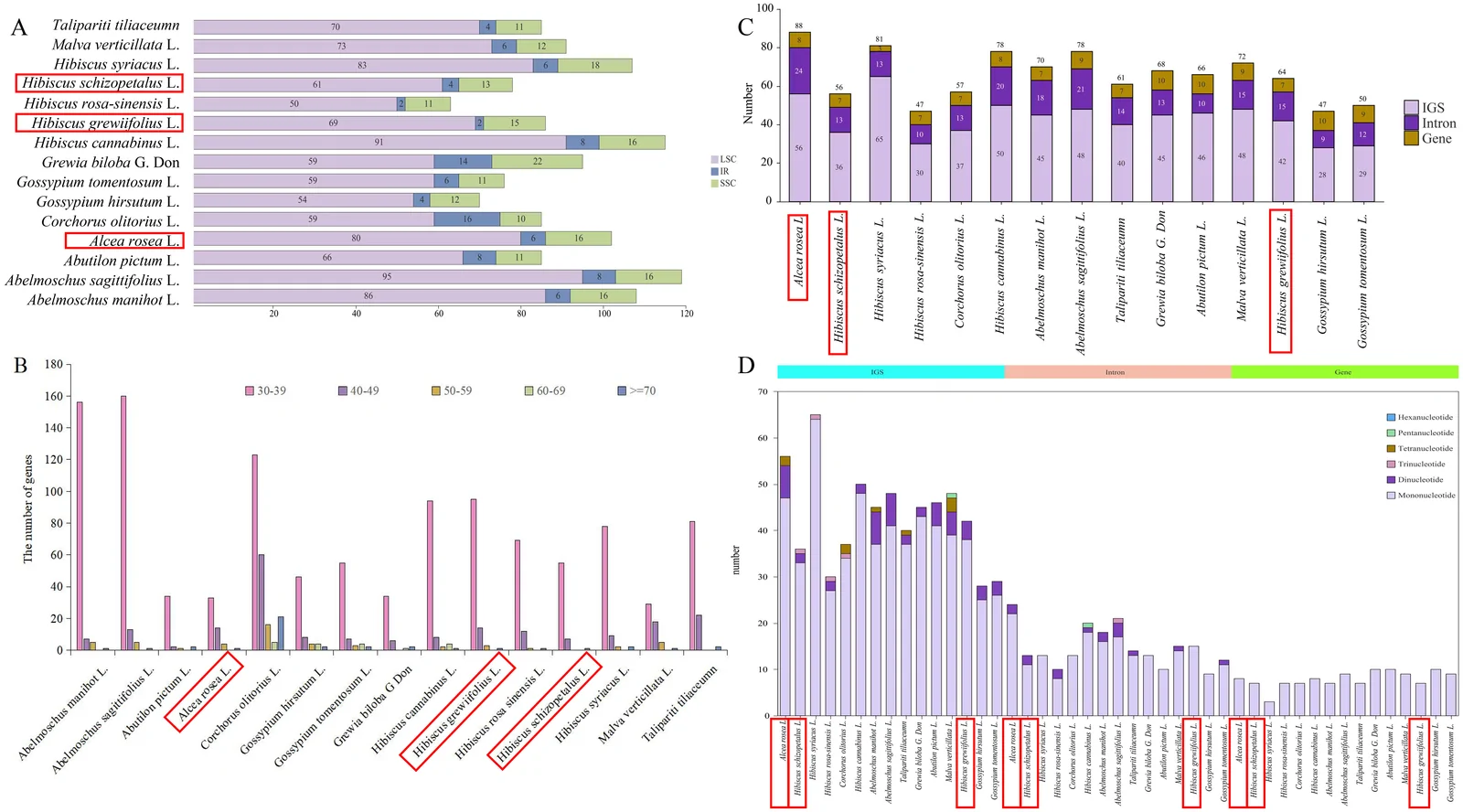
The types and numbers of SSRs and long repeats in the CP genomes of 15 Malvaceae species. The species with names is enclosed in red boxes were those that underwent measurement of the CP genome in the present study. **(A)** Distribution of SSRs in the CP DNA of 15 alvaceae species. **(B)** Length distribution of SSRs in the CP DNA of 15 Malvaceae species. **(C)** Total SSR counts in different genomic regions (IGS, genic, and intron) of the examined CP genomes. The number of SSRs detected in the IGS regions, coding sequences (genic), and intron regions is shown for each species. **(D)** Distribution of SSR repeat types across the IGS, genic, and intron regions. SSRs were classified into mono to hexanucleotide repeats on the basis of MISA parameters (mononucleotide ≥ 10, dinucleotide ≥ 6, trinucleotide ≥ 5, tetranucleotide ≥ 4, pentanucleotide ≥ 4, hexanucleotide ≥ 4). The bar chart shows the count of each repeat type in IGS, genic, and intron regions.

To further explore the sequence variations and microevolution dynamics of CP genomes, we compared the long and simple repetitive sequences of 15 species. A total of 63 to 119 SSRs of *H*. *rosa*-*sinensis* L. to *A*. *sagittifolius* L. were identified in the CP genome, covering mononucleotide, dinucleotide, trinucleotide, tetranucleotide, pentanucleotide, and hexanucleotide repeats; most of these repeats were located in the LSC region, followed by the SSC and IR regions (**Figure 3A**). Among these 15 species, the SSR characteristics of the three newly sequenced species were as follows: A. *rosea* contained 102 SSRs, H. *grewiifolius* contained 86 SSRs, and H. *schizopetalus* contained 78 SSRs. In these three species, single nucleotide repeats were the most abundant type of SSRs, and most of the SSRs were located in the LSC region, which was consistent with the overall trend of the 15 species. Mononucleotide repeats were the most common, and as the length of the repeat unit increased, the number of corresponding SSRs decreased (**Supplementary Figure 1**). Notably, hexanucleotide repeats were detected only in *H*. *schizopetalus*, *C*. *olitorius*, *G*. *hirsutum*, *A*. *sagittifolius* and *A*. *pictum*. We also identified 39 (*A*. *pictum*) to 225 (*C*. *olitorius* L.) long repeat sequences with lengths ≥ 30 bp (**Supplementary Figure 2**). They were divided into forward (F), palindrome (P), reverse (R), and complement (C) repeats, and F and P repeats were the most common. We compared the total number of SSRs in the IGS region, gene, and intron regions of the CP genomes of each tested species. The results showed that the number of SSRs in the IGS was the highest, while that in the gene region was the lowest (**Figure 3C**). The distribution of SSRs of single nucleotides to hexanucleotides in the IGS, gene, and intron regions was statistically analyzed. This analysis further confirmed that the IGS region was enriched with the majority of SSRs, while the number of SSRs in the gene region was relatively small, and they were mainly composed of multi-nucleotide repeats (**Figure 3D**). R repeat sequences were only absent in *A*. *pictum* L. and *M*. *verticillata* L. In terms of the length distribution, these repetitive segments were mainly between 30 and 39 base pairs. Sequences longer than 60 base pairs were relatively rare (**Figure 3B**). All species had repetitive sequences of ≥70 bp, but repetitive sequences with lengths of 60–69 bp were only present in *C*. *olitorius* L., *G*. *hirsutum* L., *G*. *tomentosum* L., *G*. *biloba* G. Don, and *H*. *cannabinus* L. Overall, these conserved repetitive regions provided stable markers for studying the family-level phylogeny of the selected taxa of Malvaceae, while their specific regions, especially those with abnormal numbers or unique types of repetitive sequences, provided crucial molecular clues and potential highly variable markers for distinguishing genera and even species, and could help explore the evolutionary dynamics of the genomes in each lineage.

### 3.4 Comparative genomics analysis

**Figure 4:**
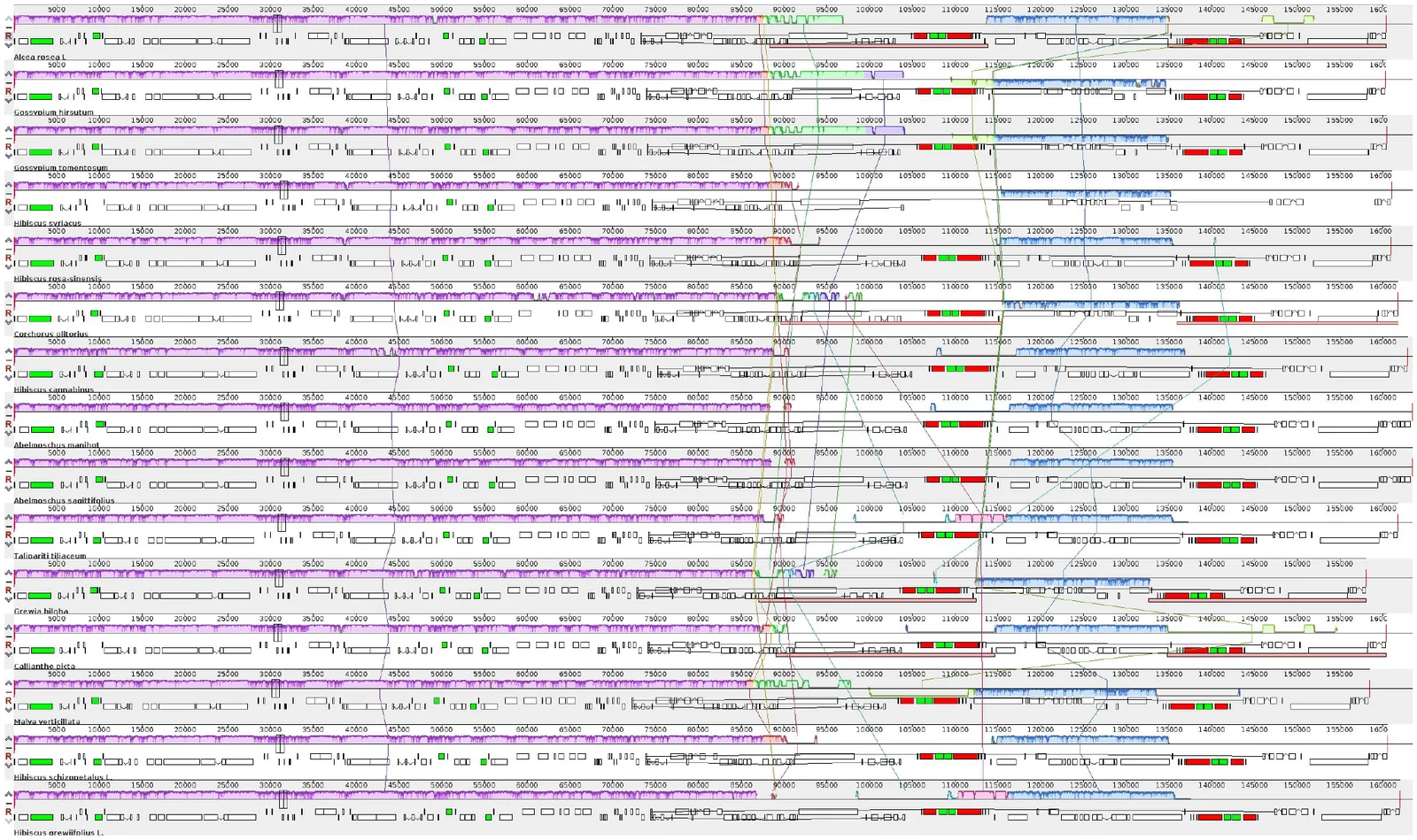
Collinearity analysis of CP DNA of 15 Malvaceae plants. Blocks of the same color are connected by lines, indicating conserved gene fragments between different species. The small blocks below the bands represents a single genes, the white blocks represents the coding genes; and the thin lines represents the introns. Green and red blocks represent tRNA and rRNA genes, respectively.

To investigate the evolutionary relationships among species in the selected taxa of Malvaceae at the genomic level, we conducted a global genomic collinearity comparison. The results showed that the CP genomes of all the compared species exhibited extremely high collinearity. The majority of homologous gene clusters showed a linear, orderly and directionally consistent arrangement among species, and no large-scale rearrangement, inversion or translocation events were detected (**Figure 4**). The CP genome sizes of the 15 Malvaceae species analyzed in this study ranged from 158,064 bp (*G. biloba* G. Don) to 163,453 bp (*A. sagittifolius*). Among them, the three newly sequenced species exhibited genome sizes of 160,403 bp (*A. rosea*), 160,493 bp (*H. schizopetalus*), and 161,978 bp (*H. grewiifolius*).

### 3.5 Comparative genomic and nucleotide polymorphism analysis

**Figure 5:**
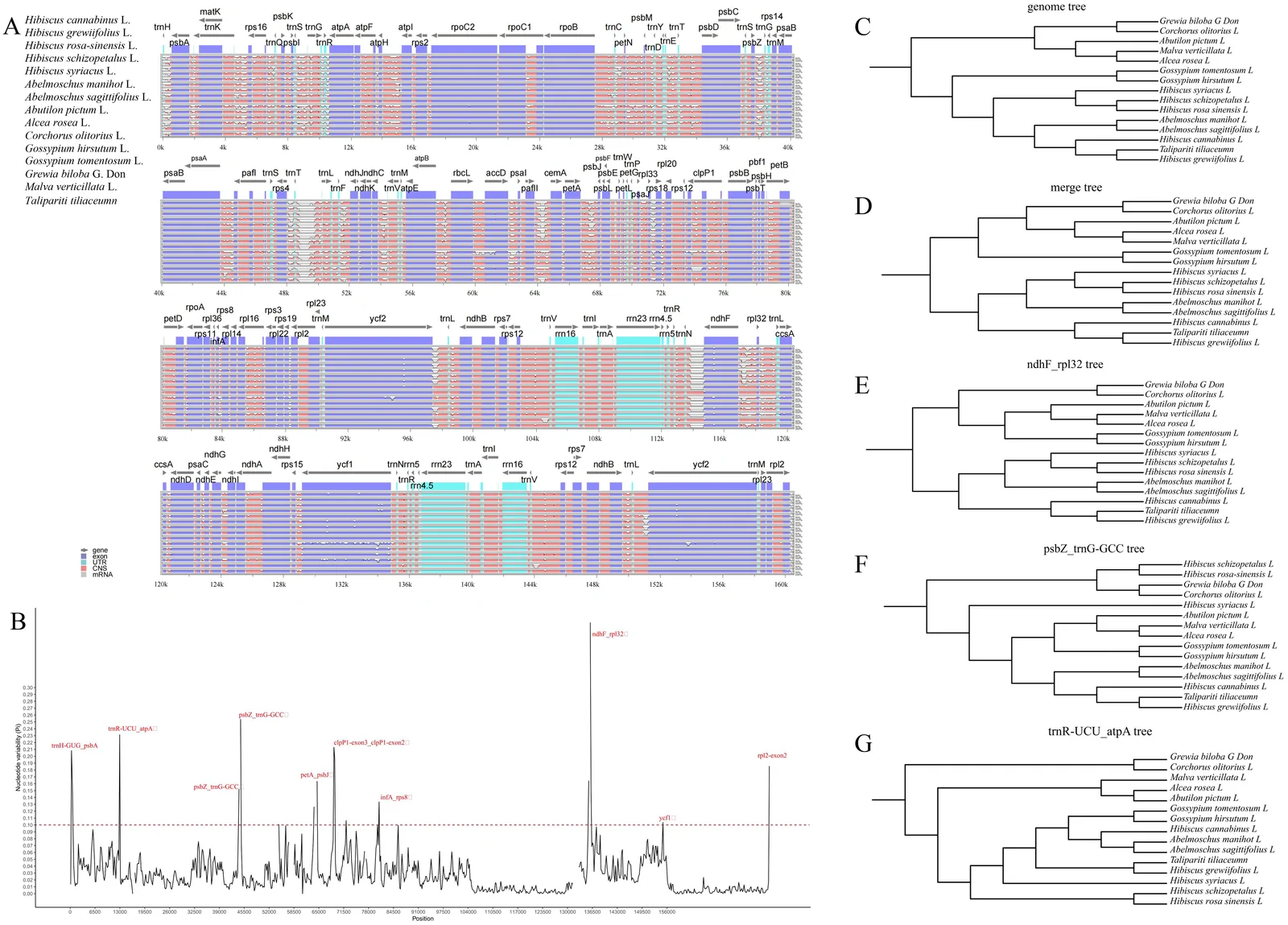
Analysis of sequence alignment and nucleotide polymorphism of the CP genomes of 15 Malvaceae species. **(A)** Collinearity analysis of the CP DNAs of 15 Malvaceae species. The parallel bars of different colors in the figure clearly show the location and distribution density of multiple key genes in the genome. Block of the same color, connected by lines, indicate conserved gene segments across different species. Small blocks beneath the color bands represent individual genes, with white blocks indicating coding genes and thin lines representing introns. Green blocks and red blocks indicate tRNA and rRNA genes, respectively. From top to bottom, the bands represent *H*. *cannabinus*, *H*. *grewiifolius*, *H*. *rosa*-*sinensis*, *H*. *schizopetalus*, *H*. *syriacus*, *A*. *manihot*., *A*. *sagittifolius*, *A*. *pictum*, *A*. *rosea*, *C*. *olitorius*, *G*. *hirsutum*, *G*. *tomentosum*, *G*. *biloba*, Don, *M*. *verticillata*, and *T*. *tiliaceumn*. **(B)** Nucleotide diversity (Pi) analysis across 15 Malvaceae species. **(C)** Full CP genome tree. **(D)** Tree based on the concatenated sequences of all three hypervariable regions. **(E)** Tree based on the hypervariable region ndhF-rpl32. **(F)** Tree based on psbZ-trnG-GCC. **(G)** Tree based on trnR-UCU-atpA.

Through genome-wide marker scans, we identified a large number of representative genes and functional regions in the CP genome (**Figure 5A**). The CP genome of *H*. *cannabinus* (NC_045873) was used as the reference. The markers were unevenly distributed within the 0–1600 kb range of the genome. Genes related to the photosystem as well as ribosomal RNA genes were clustered in highly conserved gene groups, while non-coding tRNA genes were mostly located in the intergenic regions. This distribution pattern reflected the basic structural organization of the functional regions of the CP genome. To accurately identify the regions with high genetic diversity in the population, we calculated the whole-genome nucleotide diversity index (Pi) and plotted the sliding window curve (**Figure 5B**). The results showed that the variation intensity across the genome fluctuated significantly, presenting a distinct peak-valley pattern. Several regions exhibited exceptionally high Pi value peaks, among which the intergenic regions *ndhF*_*rpl32*, *psbZ*_*trnG*-GCC, and *trnR*-UCU_*atpA* were particularly identified as highly variable hotspot regions. The Pi values in these regions were significantly higher than the background level of the genome, indicating that they had accumulated more neutral or nearly neutral mutations. These regions with high polymorphism were isolated and used to construct an evolutionary tree. Since Pi of the genome was is different from that of the species used in the tree construction, five evolutionary trees for Pi were constructed. One was the full genome evolutionary tree, called genome tree. Three other trees covered regions with high polymorphism, and a merge tree that combined these three regions was also constructed. Among these, the merge tree and *ndhF*_*rpl32* tree, clustered consistently with the genome tree. The other two regions did not match completely. Therefore, after verification through the evolutionary tree, the *ndhF*_*rpl32* region is the representative region of polymorphism (**Figures 5C–G**).

### 3.6 Codon usage bias

**Figure 6:**
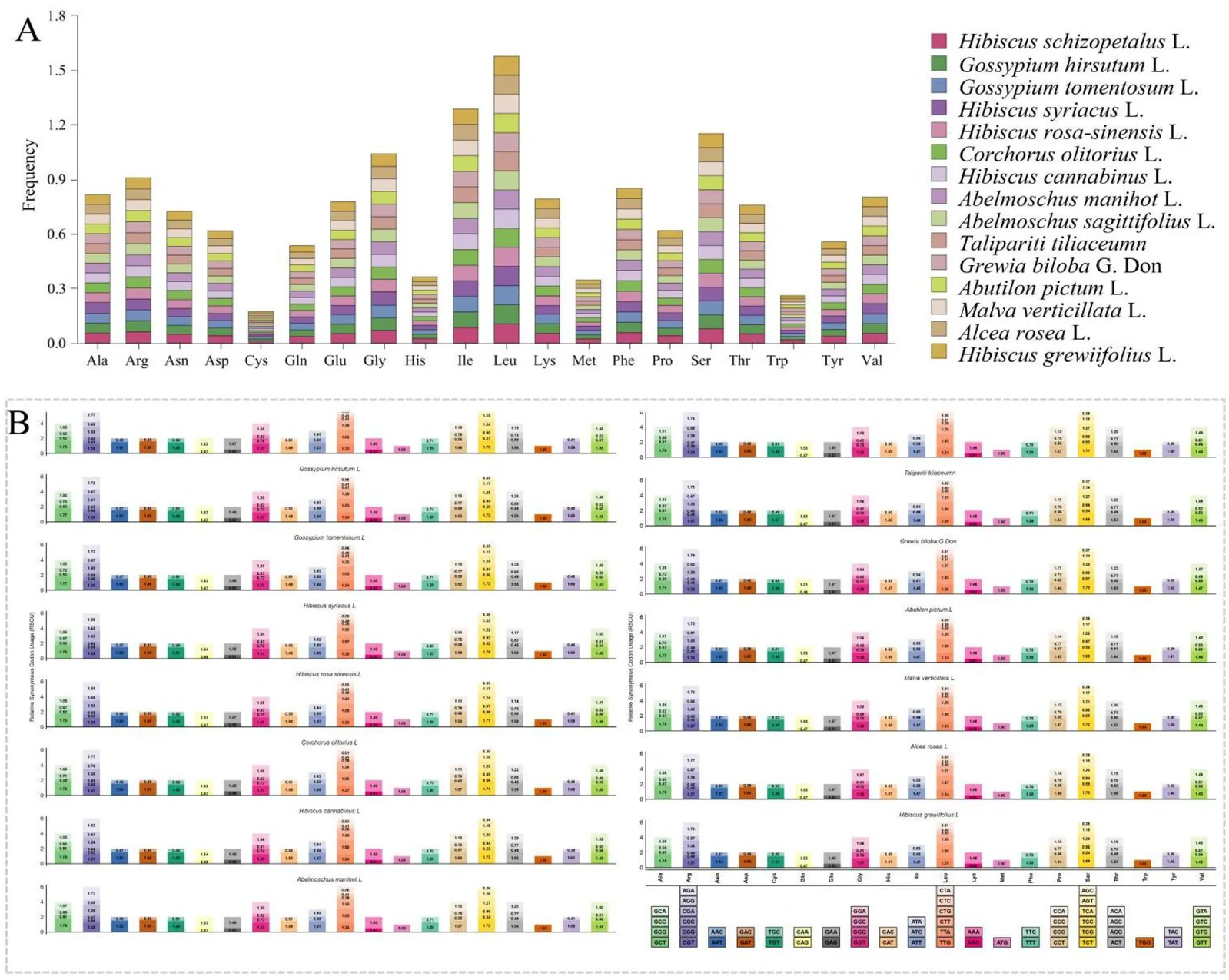
Frequency of amino acids encoded in all protein-coding genes, and the heatmap of RSCU values. **(A)** Frequency of amino acids encoded in all protein-coding genes. The x-axis represents various amino acids, while the y-axis represents the RSCU values, and different color blocks represent different species. **(B)** RSCU values for 15 Malvaceae species. The x-axis represents various amino acids, while the y-axis represents different species. The transition from green to purple indicates an increase in RSCU values, with the darker purple hues indicating a higher RSCU values.

The codon frequencies of protein-coding genes were statistically analyzed, and the bias was represented by RSCU values. An RSCU value< 1.00 indicates a lower-than-expected codon usage frequency, whereas an RSCU value > 1.00 indicates a higher-than-expected frequency. The results showed that the codon usage patterns of the 15 CP genomes were similar. The coding region contained 64 codons, including 61 codons that encoded 20 amino acids and three termination codons UGA, UAG and UAA. The codon encoding leucine had the highest frequency, while that encoding cysteine had the lowest frequency (**Figure 6A**). Methionine (ATG) and tryptophan (TGG) were each encoded by a single specific codon, so they showed no bias and their RSCU values were 1. Leucine, arginine, and serine were encoded by six synonymous codons each, while the other amino acids were encoded by two to four different codons. Among all the codons, 30 had RSCU values less than 1, while 29 had RSCU values greater than 1 (**Figure 6B**). Furthermore, among for the codons with RSCU values less than 1, 11 ended with A/U and 19 ended with G/C. In the protein-coding genes of the CP genome, the used codons showed a greater tendency to end with G/C.

**Figure 7:**
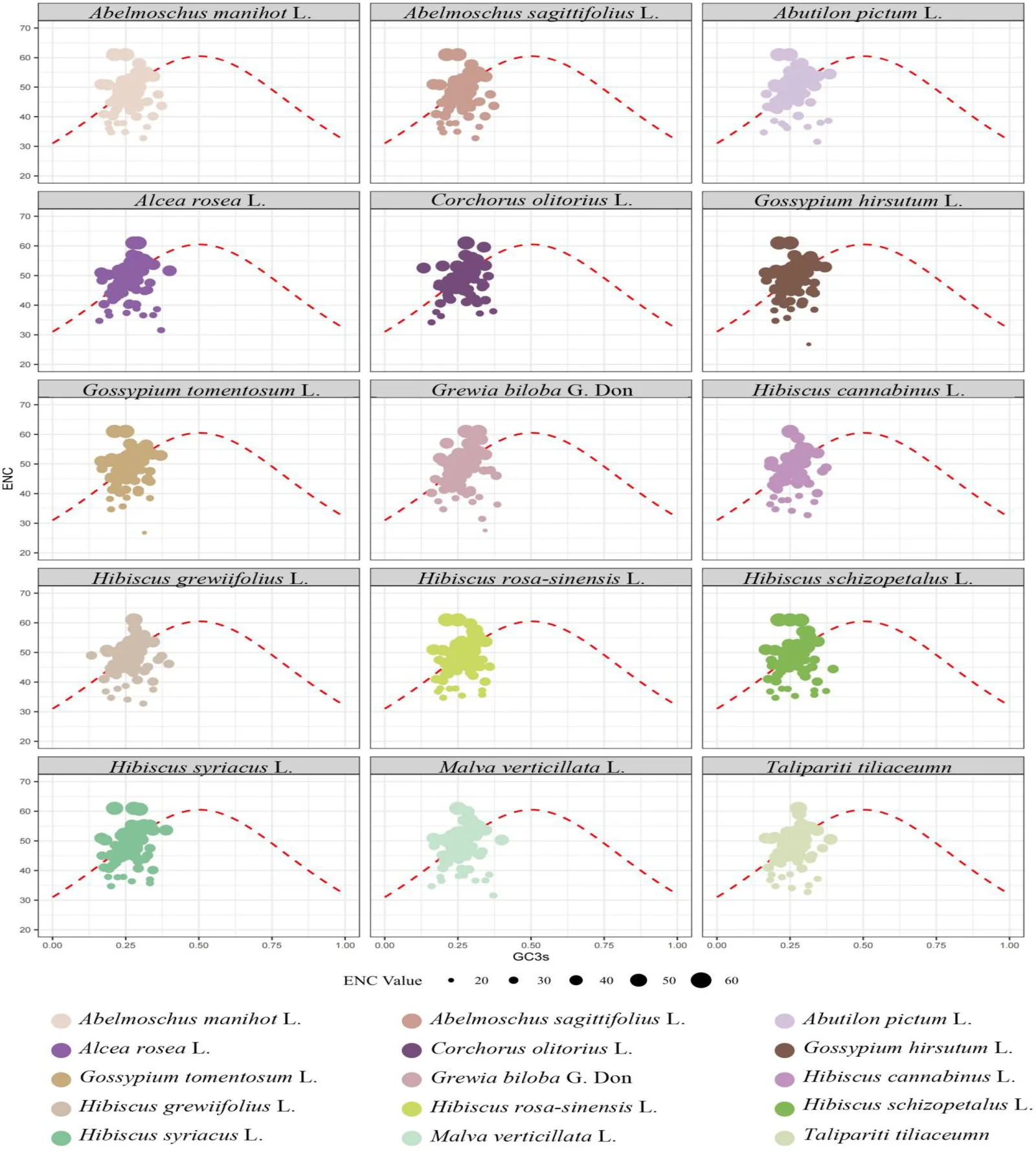
ENC plot analysis of the CP genomes of 15 Malvaceae species. Different colors represent different species. The red dotted line indicates the reference line of theoretical values, which represents the theoretical relationship between GC3s and ENCs. Larger points denote higher ENC values. Each facet corresponds to one species.

To further investigate the relationship between nucleotide composition and codon usage preferences, we employed ENC diagrams to explore the factors influencing codon usage. The ENC values of CP DNA ranged from 20.00 to 61.00, indicating that codon usage preference was generally weak. However, the patterns of gene distribution differed significantly among different species, which revealed the varied degrees of the relative contributions of mutational pressure and natural selection. If the use of codons was driven entirely by mutational pressure, the data points should be distributed along the theoretical curve in the graph. The results showed that the genes of *A*. *rosea* were closely located on the theoretical curve, indicating that the use of its codons was almost entirely dominated by mutational pressure. The gene distribution of *H*. *syriacus* L. was relatively scattered, but mainly concentrated near the curve, showing transitional characteristics. These characteristics were mainly influenced by mutational pressure, and natural selection initially also played a certain role. In sharp contrast, the majority of the *H*. *rosa*-*sinensis* L. genes deviated significantly and concentrated below the theoretical curve, clearly indicating the crucial role of strong natural selection in their codon optimization. These results indicated that the codon usage preferences of the CP genomes in the selected taxa of Malvaceae were influenced by both mutational pressure and natural selection, but the relative weights of these factors varied among different species (**Figure 7**).

### 3.7 Analysis of selection pressure

**Figure 8:**
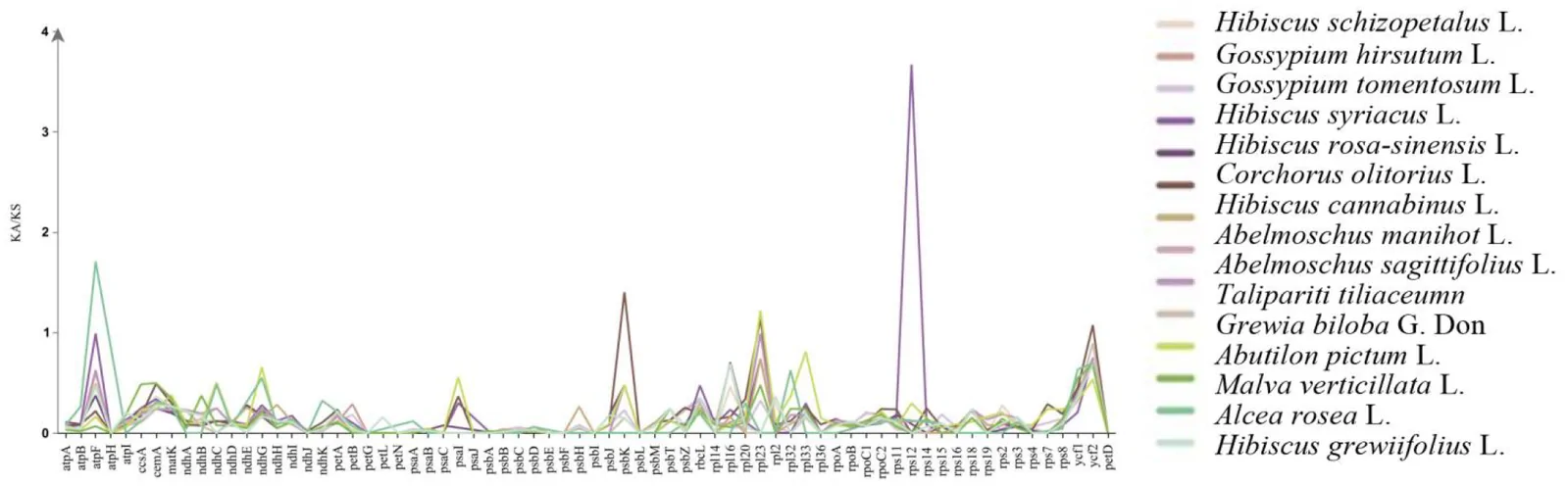
Selection pressure analysis of CP DNA genes in 15 Malvaceae plants. The x-axis represents a single gene, and the y-axis represents the Ka/Ks value. The lines of each color represent a species.

To further compare the natural selection pressure on protein-coding genes among different species, we calculated the ratio of nonsynonymous and synonymous substitution rates (Ka/Ks) (**Figure 8**). The analysis revealed that all species exhibited a similar trend in the distribution of polymorphisms, with lower values in conserved regions, indicating that these genes were under strong purifying selection constraints. However, this proportion was elevated in several highly variable regions. Most importantly, in the *trnH*-*psbA* region, the Ka/Ks ratio of *H. syriacus* L. showed a significant peak of approximately 4.0, which robustly indicates that the gene may have undergone positive selection in *H. syriacus* L. species, exhibiting adaptive evolution. This discovery was in line with the results of the Pi value analysis, which identified this area as a hotspot of high variability. However, it revealed different potential evolutionary forces, implying that a high Pi value may be the result of neutral mutation accumulation or the population history while a high Ka/Ks ratio directly pointed to adaptive selection pressure.

### 3.8 Phylogenetic analysis of plants in Malvaceae

**Figure 9:**
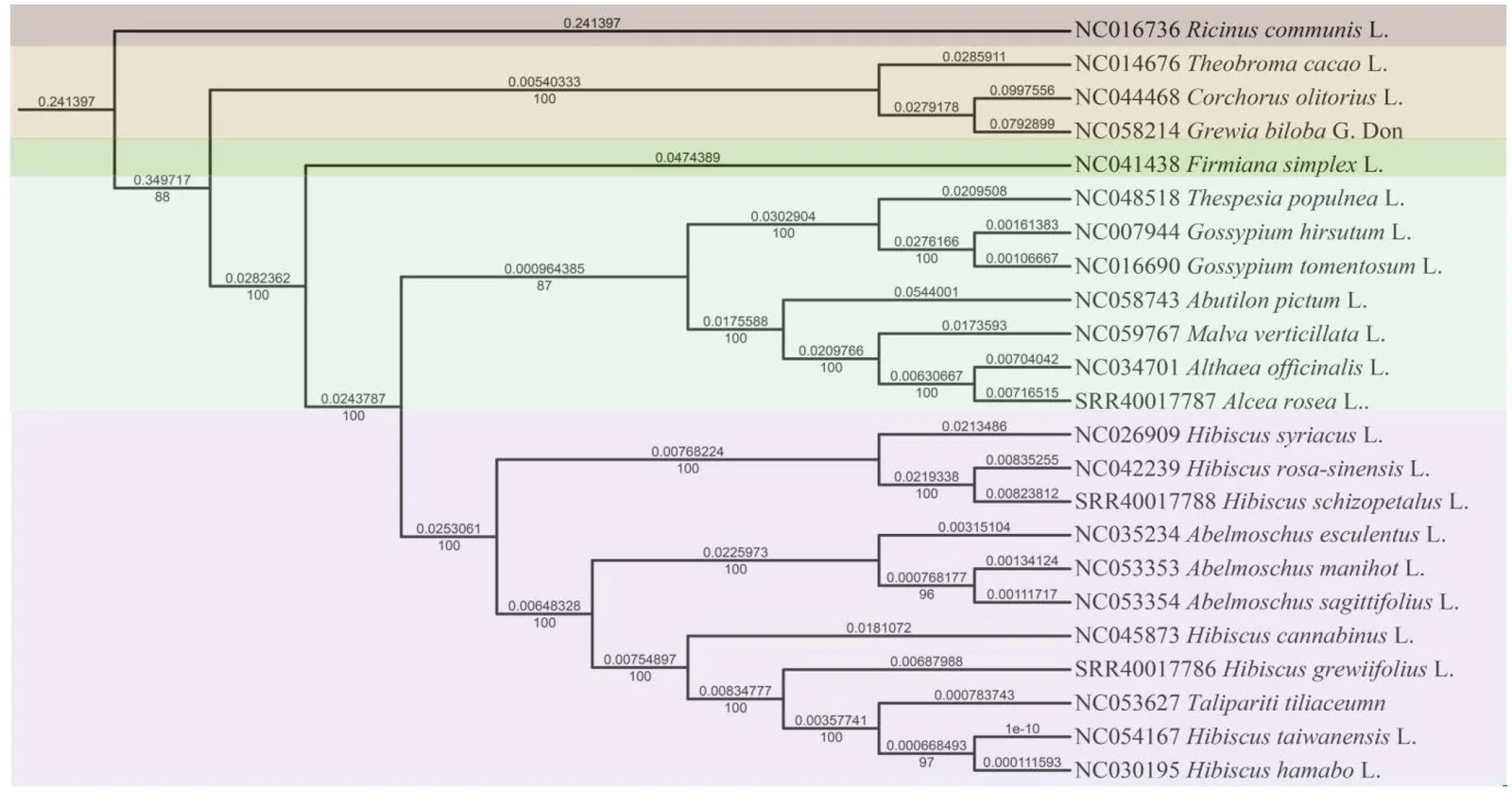
Phylogenetic tree based on SNPs in whole CP genomes of 23 species constructed using the ML method.

To explore the species classification and genetic evolutionary relationships of plants in the selected taxa of Malvaceae, SNPs in whole CP genomes data of the newly assembled three CP genomes and 20 downloaded CP genomes covering 13 genera were analyzed. A phylogenetic tree was constructed using *R. communis* L. as the outgroup (**Figure 9**). The phylogenetic tree divided all 23 species into five main branches (groups I-V). Among them, Group I corresponded to the outgroup *R*. *communis*. *Theobroma cacao* L., *G. biloba* G. Don and *C. olitorius* formed the most basal Group II, which showed the most distant genetic relationship with other species. *Firmiana simplex* L. singly formed Group III; the remaining species formed groups IV-V, which represented the core Malvaceae clade. Group IV included *Thespesia populnea* L., *G. hirsutum*, *G. tomentosum*, *A. pictum*, *M. verticillata*, *Althaea officinalis* L., and *A. rosea*. Group V contained all species of the genera *Hibiscus* and *Abelmoschus*. Among them, *H. schizopetalus*, *H. syriacus*, and *H. rosa-sinensis* formed a subclade, while *H*. *grewiifolius* was located in another relatively independent subclade. This further supported the revised classification proposal of incorporating genus *Abelmoschus* into genus *Hibiscus*. Additionally, the basal positions of Group II and Group III were largely consistent with the distribution of the traditional definitions of the Sterculioideae and Grewioideae subfamilies in the Malvaceae family, suggesting that the phylogenetic framework constructed in this study had good consistency with the existing family-level classification system. In conclusion, the complete CP genome provided greater discriminatory power than individual hypervariable regions for species identification and may serve as a super-barcode for the accurate identification of *Hibiscus* and related species. This phylogenetic framework provides a reliable genomic basis for revising the classification and species identification of *Hibiscus* and its related species.

## 4 Discussion

Although molecular systematics research has facilitated the classification of the Malvaceae family to some extent, phylogenetic trees constructed on the basis of a few DNA fragments often have low support rates at the interspecific and intraspecific (closely related species) levels due to insufficient information sites. In particular, the relationships between *Abelmoschus* and the *Hibiscus* as well as the boundaries between tribes have remained topics of debate. Moreover, comparative genomics research on CP genomes in the Malvaceae family is still relatively scarce, and the lack of high-variability molecular markers for systematic identification has restricted precise identification of species in this family and the analysis of deep phylogenetic relationships (Li D. et al., 2025). In response to these knowledge gaps, three representative species, namely, *A. rosea*, *H. schizopetalus* and *H. grewiifolius*, were selected in this study. Their whole CP genomes were sequenced, assembled and annotated, and systematic comparative genomics analysis was performed. The aim of this study was to provide crucial molecular evidence at the genomic level. A detailed discussion covering several aspects, including genomic structural characteristics, dynamic IR/SC boundaries, nucleotide polymorphisms, and phylogenetic relationships, was conducted.

### 4.1 Basic characteristics of CP genomes in Malvaceae species

These CP genomes of the three Malvaceae species evaluated in this study showed a classical quadripartite circular structure composed of a pair of IRs to separate the LSC and the SSC regions (Bai et al., 2023). These genomes were highly similar in length, GC content, gene composition and number, and codon usage patterns, reflecting the overall conservation of CP genomes during long term evolution (Harris et al., 2013; Williams et al., 2019; Robbins and Kelly, 2023). Codon preference analysis further showed that leucine was the most frequently used amino acid, which was consistent with the results for most terrestrial plants and confirmed the mature and conserved translation mechanism of CP genome (Somaratne et al., 2020; Li D. et al., 2025). The overall structural stability of the CP genomes provided a reliable framework for cross-species genome comparison, identification of variable sites, and phylogenetic reconstruction. It also demonstrated that the CP genome is suitable for higher-level classification and species distinction within the Malvaceae family.

### 4.2 IR/SC boundary dynamics and genomic length variations

Under the conservation of the macro structure, the local regions of the genome show significant dynamic evolutionary characteristics. The IR/SC analysis revealed that the overlap length difference of JLB boundary among different species was up to 908 bp, which was the main structural factor causing variations in the CP genome length. This phenomenon is consistent with the findings in other plant groups, indicating that the dynamic contraction and expansion of the IR boundary is one of the common driving forces of CP genome evolution (Lu and Luo, 2022). Notably, the instability of this boundary was associated with the distribution of SSRs and long repeats in the genome. SSRs and long repeats are important genetic markers in population genetics and evolution (Palmer and Thompson, 1982). The SSRs identified in this study were mainly single nucleotide repeats, and the long repeat sequences were mostly forward and palindromic repeats. The distribution characteristics of these repetitive sequences may be closely related to the contraction/expansion events of the IR boundary, and jointly participated in the regulation of the size evolution of the CP genome (Wu et al., 2021).

### 4.3 Comparative genomics and nucleotide polymorphisms

DNA barcoding technology has become an important tool for species identification. Common fragments such as *matK, rbcL* and *trnH-psbA* are widely used in this technology (Skuza et al., 2019). However, these fragments often face limitations associated with insufficient resolution in the identification of close relative species. On the basis of the CP genome-wide comparison and nucleotide diversity (Pi) analysis, this study revealed a non-uniform distribution pattern of variations in the genome, wherein highly variable hotspots were significantly enriched in the non-coding spacers of the LSC and SSC regions, while the IR and coding regions were relatively conserved. This distribution pattern is consistent with the finding of previous studies (Wang et al., 2014; Wang et al., 2015). Notably, the *ycf1* gene, which is located near the IR/SSC boundary, showed significant length polymorphism and sequence variation. Although the design of its full-length primers was difficult, some of its high-variable regions showed outstanding species identification potential (Rozas et al., 2017; Li et al., 2023). On the basis of these findings, this study proposed to combine the hypervariable region of the *ycf1* gene with hypervariable spacers such as *ndhF* and *rpl32* to construct a “super barcode” suitable for identification of relatives and cultivars of the selected taxa of Malvaceae (Weixue et al., 2026). This strategy is based on genome-wide information to screen specific hypervariable regions, which shows higher resolution than traditional barcodes. This approach has been successfully applied in *Camellia* L. and *Illicium* L. and provides a new feasible path for accurate identification of species belonging to selected taxa of Malvaceae species (Yu et al., 2021).

### 4.4 Selection pressure analysis and evolutionary implications

Relationships are subject to strong purifying selection constraints. Purifying selection can eliminate harmful mutations and stabilize the core gene sequences related to photosynthesis and energy metabolism, ensuring the stability of the basic physiological functions of the CPs. This is also the fundamental reason for the overall high conservation of the CP coding regions. The sequence stability under this functional constraint limits the ability of a single conventional DNA barcode to provide sufficient variation sites among closely related species. In contrast, comparisons at the SNPs in whole CP genomes level can effectively integrate the information of high-variable regions, thereby providing sufficient molecular evidence for phylogenetic reconstruction. The phylogenetic tree constructed on the basis of the complete CP genome sequence clearly analyzed the evolutionary relationships between the three Malvaceae species and their related species with extremely high support rates. The analysis results clearly showed that the *Abelmoschus* species were nested within the Hibiscus clade with high support values. This strong molecular systematic evidence supports the inclusion of *Abelmoschus* in *Hibiscus (*Bai et al., 2023). Unlike previous studies based on limited gene fragments that often yielded insufficient support rates (Baum et al., 2004; Wang et al., 2023; Geng et al., 2024), the use of the complete genome data to construct the main nodes of the phylogenetic framework in this study yielded extremely high support rates, which significantly improved the reliability of the topological structure. This highly reliable phylogenetic tree not only clarified the long-standing intergeneric relationships but also provided a solid molecular systematic foundation for future studies of adaptive evolution and population genetics in Malvaceae.

## 5 Conclusions

This study obtained the complete CP genomes of three Malvaceae plants, *A*. *rosea*, *H*. *schizopetalus*, and *H*. *grewiifolius*, through sequencing, assembly, and annotation. In combination with 20 published CP genomes of the Malvaceae family, these three genomes were used to conduct systematic comparative genomics analyses, repeat-sequence analysis, nucleotide-polymorphism analysis, and phylogenetic reconstruction. The results indicated that the CP genomes of these three Malvaceae species and their close relatives were highly conserved in terms of gene composition, sequence, and overall structure but showed marked contraction and expansion at the IR/SC boundaries, which were the primary drivers of genome length variation. SSR and long repetitive sequences were enriched in the LSC and IGS regions, with mononucleotide repeats, forward repeats, and palindrome repeats being the main types. Nucleotide-polymorphism analysis identified multiple highly variable regions, which could serve as potential super barcode candidate markers for precise identification of closely related species of the Malvaceae family. Phylogenetic analysis resolved the phylogenetic relationships of the Hibiscus genus and its related groups with high support rates and clearly supported the classification revision proposal of including *Abelmoschus* into *Hibiscus*. This study not only enriched the CP genome resources of the Malvaceae family, but also provided a reliable genomic foundation for species identification, classification revisions, and molecular marker development in this family.

## Data Availability

The datasets presented in this study can be found in online repositories. The names of the repository/repositories and accession number(s) can be found in the article/**Supplementary Material**.
