# Correction: Comparative genomics and phylogenetic analysis of three Malvaceae species on the basis of chloroplast genomes

**DOI:** 10.3389/fpls.2026.1987667

**Published:** 2026-09-21

**Authors:** Frontiers Production Office

**Affiliations:** Frontiers Media SA, Lausanne, Switzerland

**Keywords:** chloroplast genome, hypervariable regions, Malvaceae, phylogenetic analysis, phylogenomics

The affiliation of Reviewer “Dilnur Tussipkan” has been corrected to “National Center for Biotechnology, Kazakhstan”.

The original version of this article has been updated.

