# Supplementary material for: Comparative genomics and phylogenetic analysis of three Malvaceae species on the basis of chloroplast genomes
Source: Front Plant Sci. 2026 Aug 31;17:1860109. doi: 10.3389/fpls.2026.1860109 (PMC13572635; doi:10.3389/fpls.2026.1860109)
Supplement: Supplementary file 1 [file DataSheet1.docx]

Figure S1. Types of SSRs in the cpDNA of 15 Malvaceae species. The x-axis represents different species, the y-axis represents the number, and the color represents different types.

Figure S2. Types and quantities of LTR sequences in the cpDNA of 15 Malvaceae species. The x-axis represents different varieties, the y-axis represents the length of the repeat sequence, and different colors represent forward (F), palindrome (P), reverse (R) and complement (C).
